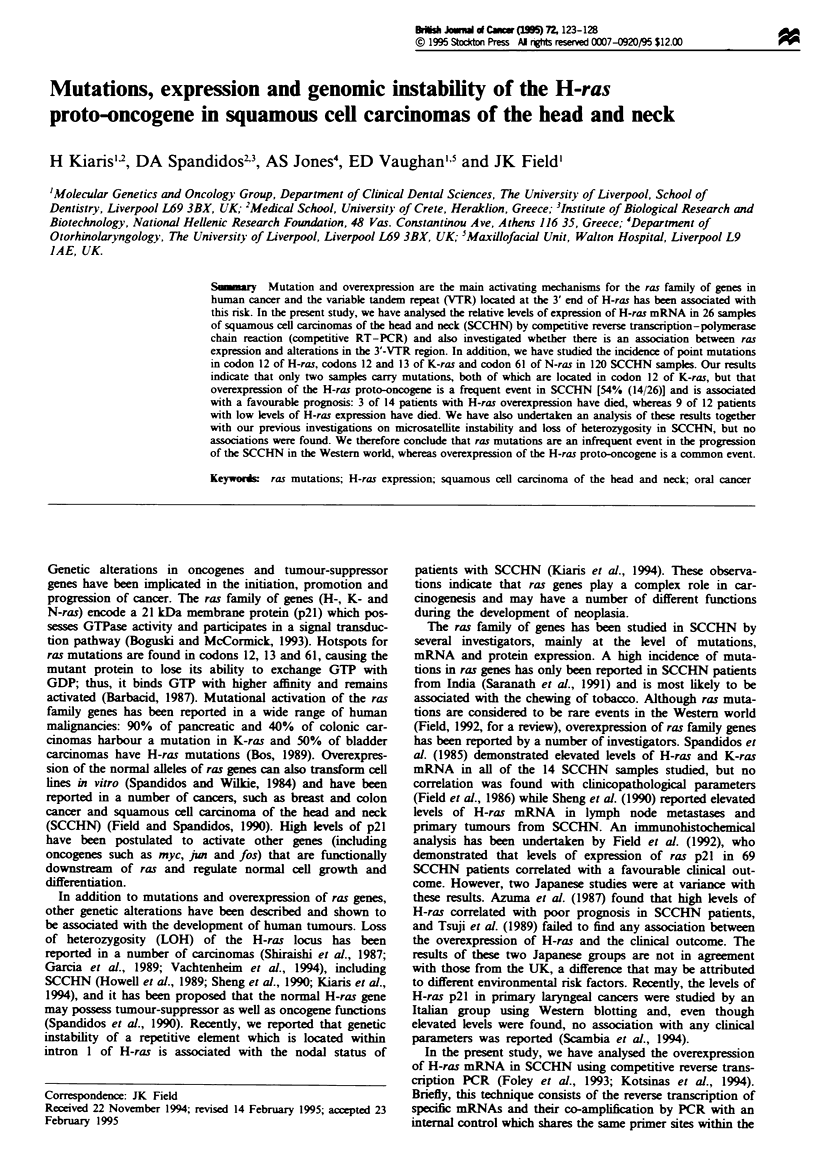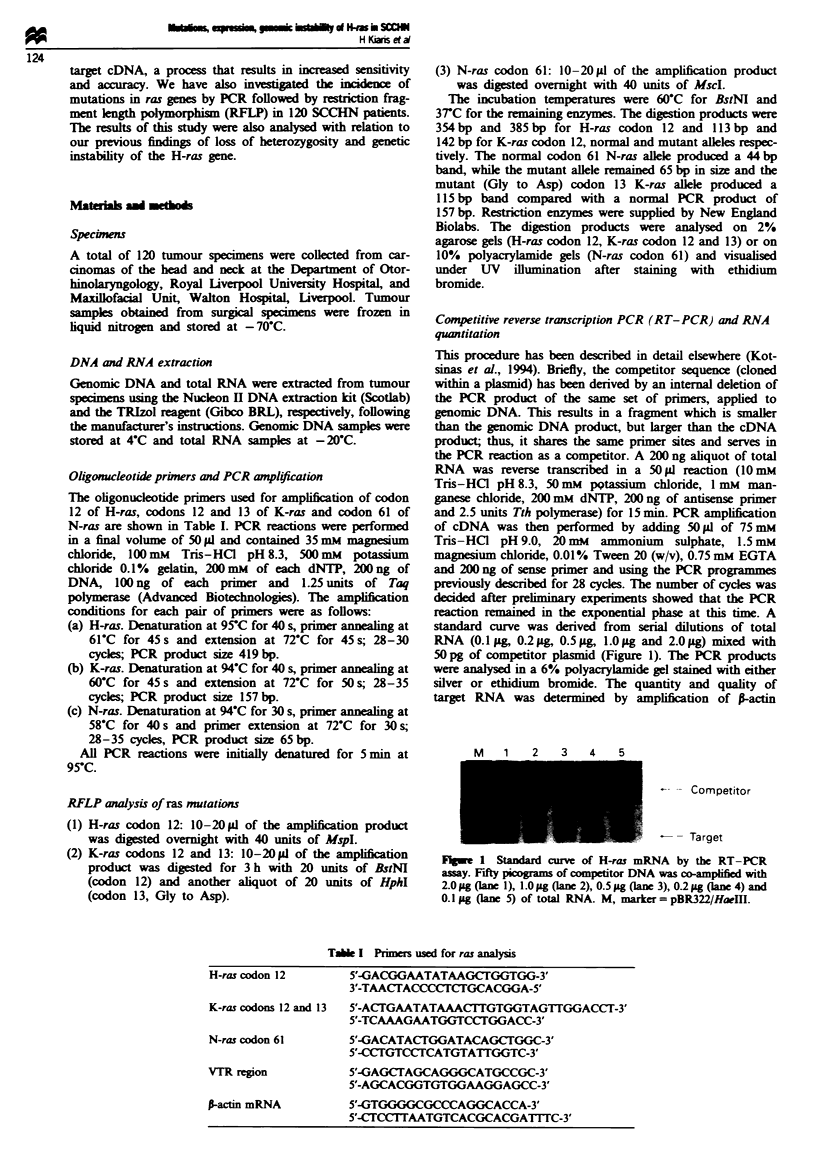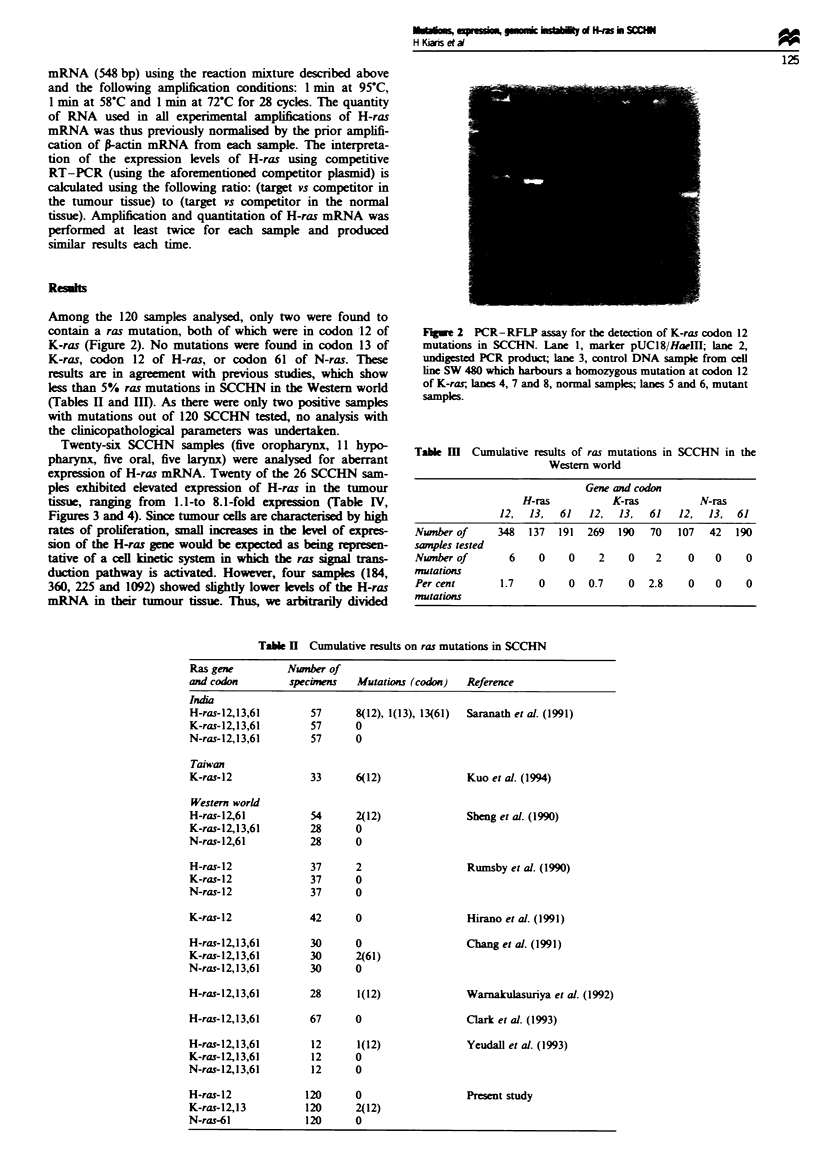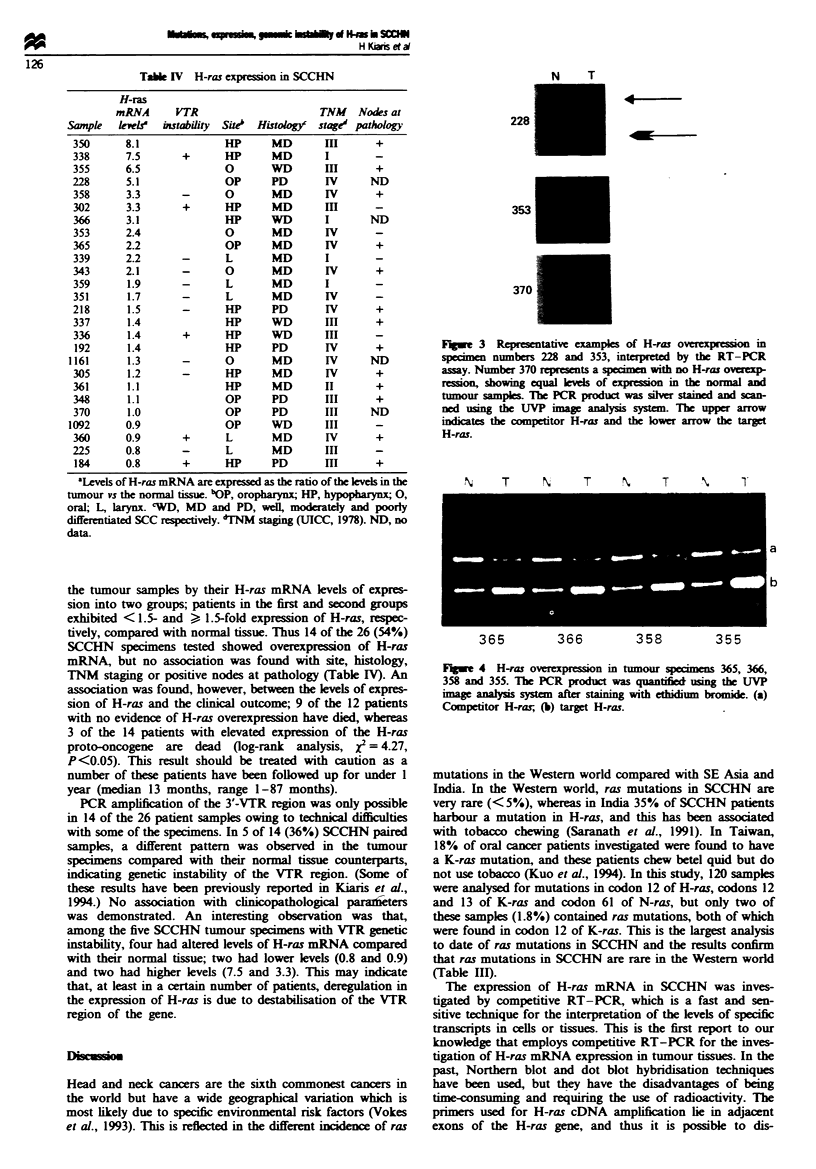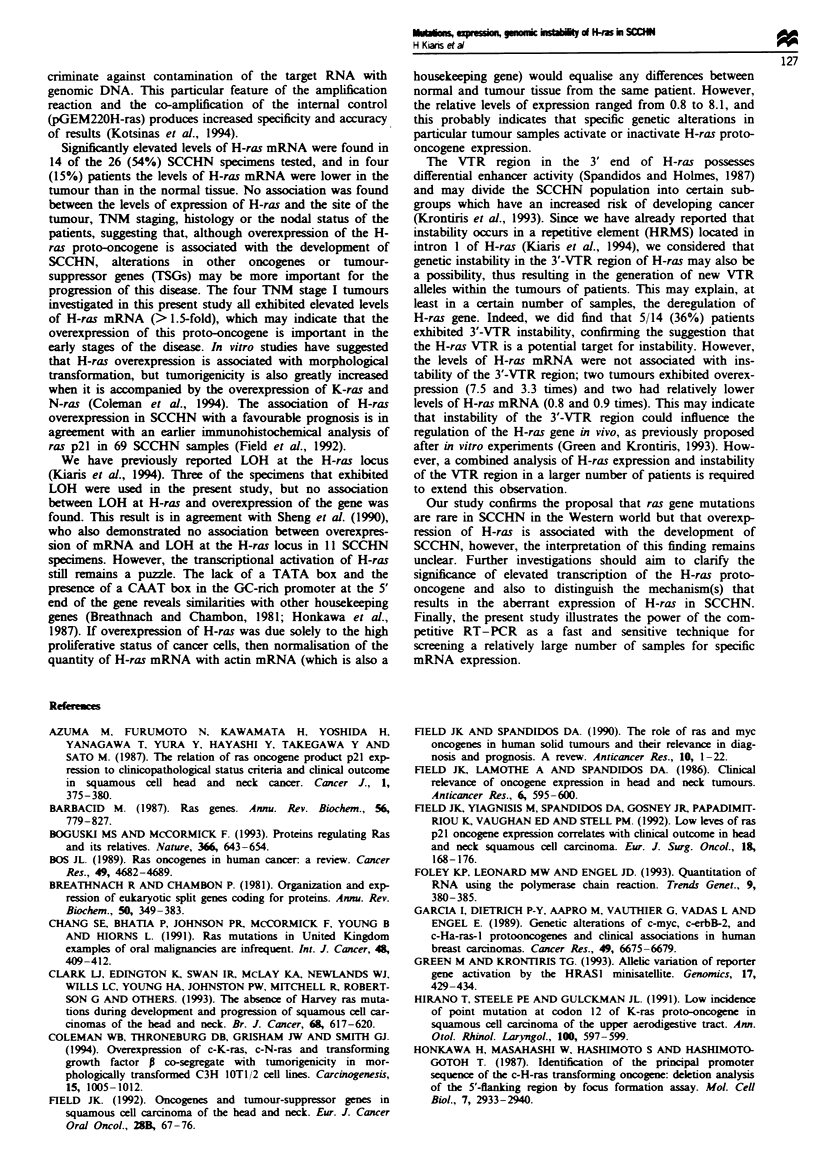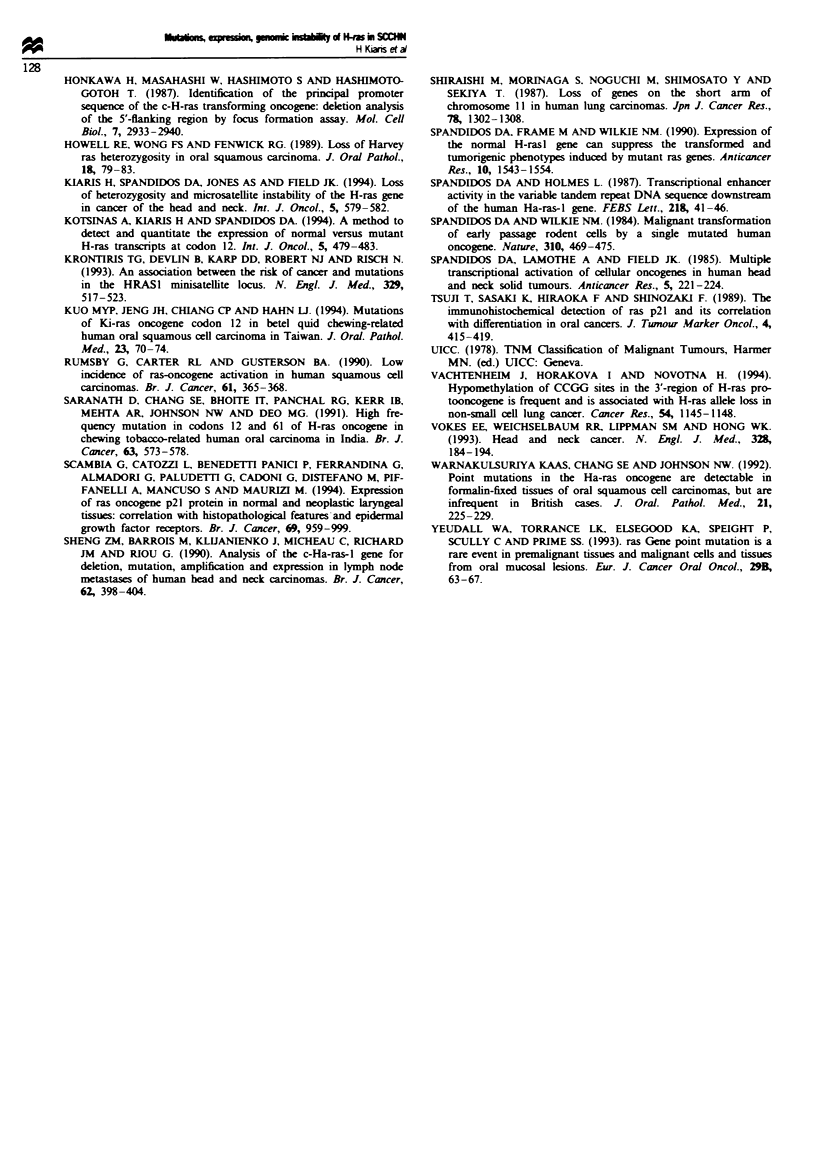# Mutations, expression and genomic instability of the H-ras proto-oncogene in squamous cell carcinomas of the head and neck.

**DOI:** 10.1038/bjc.1995.287

**Published:** 1995-07

**Authors:** H. Kiaris, D. A. Spandidos, A. S. Jones, E. D. Vaughan, J. K. Field

**Affiliations:** Department of Clinical Dental Sciences, University of Liverpool, School of Dentistry, UK.

## Abstract

**Images:**


© 1995 Stockton Press All rights reserved 0007-0920/95 $12.00

### Mutations, expression and genomic instability of the H-*ras* proto-oncogene in squamous cell carcinomas of the head and neck

H Kiaris[1,2], DA Spandidos[2,3], AS Jones[4], ED Vaughan[1,5] and JK Field[1]

[1]*Molecular Genetics and Oncology Group, Department of Clinical Dental Sciences, The University of Liverpool, School of Dentistry, Liverpool L69 3BX, UK;* [2]*Medical School, University of Crete, Heraklion, Greece;* [3]*Institute of Biological Research and Biotechnology, National Hellenic Research Foundation, 48 Vas. Constantinou Ave, Athens 116 35, Greece;* [4]*Department of Otorhinolaryngology, The University of Liverpool, Liverpool L69 3BX, UK;* [5]*Maxillofacial Unit, Walton Hospital, Liverpool L9 1AE, UK.*

**Summary** Mutation and overexpression are the main activating mechanisms for the *ras* family of genes in human cancer and the variable tandem repeat (VTR) located at the 3′ end of H-*ras* has been associated with this risk. In the present study, we have analysed the relative levels of expression of H-*ras* mRNA in 26 samples of squamous cell carcinomas of the head and neck (SCCHN) by competitive reverse transcription-polymerase chain reaction (competitive RT-PCR) and also investigated whether there is an association between *ras* expression and alterations in the 3′-VTR region. In addition, we have studied the incidence of point mutations in codon 12 of H-*ras*, codons 12 and 13 of K-*ras* and codon 61 of N-*ras* in 120 SCCHN samples. Our results indicate that only two samples carry mutations, both of which are located in codon 12 of K-*ras*, but that overexpression of the H-*ras* proto-oncogene is a frequent event in SCCHN [54% (14/26)] and is associated with a favourable prognosis: 3 of 14 patients with H-*ras* overexpression have died, whereas 9 of 12 patients with low levels of H-*ras* expression have died. We have also undertaken an analysis of these results together with our previous investigations on microsatellite instability and loss of heterozygosity in SCCHN, but no associations were found. We therefore conclude that *ras* mutations are an infrequent event in the progression of the SCCHN in the Western world, whereas overexpression of the H-*ras* proto-oncogene is a common event.

**Keywords:** *ras* mutations; H-*ras* expression; squamous cell carcinoma of the head and neck; oral cancer

Genetic alterations in oncogenes and tumour-suppressor genes have been implicated in the initiation, promotion and progression of cancer. The *ras* family of genes (H-, K- and N-*ras*) encode a 21 kDa membrane protein (p21) which possesses GTPase activity and participates in a signal transduction pathway (Boguski and McCormick, 1993). Hotspots for *ras* mutations are found in codons 12, 13 and 61, causing the mutant protein to lose its ability to exchange GTP with GDP; thus, it binds GTP with higher affinity and remains activated (Barbacid, 1987). Mutational activation of the *ras* family genes has been reported in a wide range of human malignancies: 90% of pancreatic and 40% of colonic carcinomas harbour a mutation in K-*ras* and 50% of bladder carcinomas have H-*ras* mutations (Bos, 1989). Overexpression of the normal alleles of *ras* genes can also transform cell lines *in vitro* (Spandidos and Wilkie, 1984) and have been reported in a number of cancers, such as breast and colon cancer and squamous cell carcinoma of the head and neck (SCCHN) (Field and Spandidos, 1990). High levels of p21 have been postulated to activate other genes (including oncogenes such as *myc*, *jun* and *fos*) that are functionally downstream of *ras* and regulate normal cell growth and differentiation.

In addition to mutations and overexpression of *ras* genes, other genetic alterations have been described and shown to be associated with the development of human tumours. Loss of heterozygosity (LOH) of the H-*ras* locus has been reported in a number of carcinomas (Shiraishi *et al.*, 1987; Garcia *et al.*, 1989; Vachtenheim *et al.*, 1994), including SCCHN (Howell *et al.*, 1989; Sheng *et al.*, 1990; Kiaris *et al.*, 1994), and it has been proposed that the normal H-*ras* gene may possess tumour-suppressor as well as oncogene functions (Spandidos *et al.*, 1990). Recently, we reported that genetic instability of a repetitive element which is located within intron 1 of H-*ras* is associated with the nodal status of patients with SCCHN (Kiaris *et al.*, 1994). These observations indicate that *ras* genes play a complex role in carcinogenesis and may have a number of different functions during the development of neoplasia.

The *ras* family of genes has been studied in SCCHN by several investigators, mainly at the level of mutations, mRNA and protein expression. A high incidence of mutations in *ras* genes has only been reported in SCCHN patients from India (Saranath *et al.*, 1991) and is most likely to be associated with the chewing of tobacco. Although *ras* mutations are considered to be rare events in the Western world (Field, 1992, for a review), overexpression of *ras* family genes has been reported by a number of investigators. Spandidos *et al.* (1985) demonstrated elevated levels of H-*ras* and K-*ras* mRNA in all of the 14 SCCHN samples studied, but no correlation was found with clinicopathological parameters (Field *et al.*, 1986) while Sheng *et al.* (1990) reported elevated levels of H-*ras* mRNA in lymph node metastases and primary tumours from SCCHN. An immunohistochemical analysis has been undertaken by Field *et al.* (1992), who demonstrated that levels of expression of *ras* p21 in 69 SCCHN patients correlated with a favourable clinical outcome. However, two Japanese studies were at variance with these results. Azuma *et al.* (1987) found that high levels of H-*ras* correlated with poor prognosis in SCCHN patients, and Tsuji *et al.* (1989) failed to find any association between the overexpression of H-*ras* and the clinical outcome. The results of these two Japanese groups are not in agreement with those from the UK, a difference that may be attributed to different environmental risk factors. Recently, the levels of H-*ras* p21 in primary laryngeal cancers were studied by an Italian group using Western blotting and, even though elevated levels were found, no association with any clinical parameters was reported (Scambia *et al.*, 1994).

In the present study, we have analysed the overexpression of H-*ras* mRNA in SCCHN using competitive reverse transcription PCR (Foley *et al.*, 1993; Kotsinas *et al.*, 1994). Briefly, this technique consists of the reverse transcription of specific mRNAs and their co-amplification by PCR with an internal control which shares the same primer sites within the

Correspondence: JK Field
Received 22 November 1994; revised 14 February 1995; accepted 23 February 1995

target cDNA, a process that results in increased sensitivity and accuracy. We have also investigated the incidence of mutations in *ras* genes by PCR followed by restriction fragment length polymorphism (RFLP) in 120 SCCHN patients. The results of this study were also analysed with relation to our previous findings of loss of heterozygosity and genetic instability of the H-*ras* gene.

### Materials and methods

### *Specimens*

A total of 120 tumour specimens were collected from carcinomas of the head and neck at the Department of Otorhinolaryngology, Royal Liverpool University Hospital, and Maxillofacial Unit, Walton Hospital, Liverpool. Tumour samples obtained from surgical specimens were frozen in liquid nitrogen and stored at −70°C.

### *DNA and RNA extraction*

Genomic DNA and total RNA were extracted from tumour specimens using the Nucleon II DNA extraction kit (Scotlab) and the TRIzol reagent (Gibco BRL), respectively, following the manufacturer's instructions. Genomic DNA samples were stored at 4°C and total RNA samples at −20°C.

### *Oligonucleotide primers and PCR amplification*

The oligonucleotide primers used for amplification of codon 12 of H-*ras*, codons 12 and 13 of K-*ras* and codon 61 of N-*ras* are shown in Table I. PCR reactions were performed in a final volume of 50 µl and contained 35 mM magnesium chloride, 100 mM Tris–HCl pH 8.3, 500 mM potassium chloride 0.1% gelatin, 200 mM of each dNTP, 200 ng of DNA, 100 ng of each primer and 1.25 units of *Taq* polymerase (Advanced Biotechnologies). The amplification conditions for each pair of primers were as follows:

(a) H-*ras*. Denaturation at 95°C for 40 s, primer annealing at 61°C for 45 s and extension at 72°C for 45 s; 28–30 cycles; PCR product size 419 bp.
(b) K-*ras*. Denaturation at 94°C for 40 s, primer annealing at 60°C for 45 s and extension at 72°C for 50 s; 28–35 cycles; PCR product size 157 bp.
(c) N-*ras*. Denaturation at 94°C for 30 s, primer annealing at 58°C for 40 s and primer extension at 72°C for 30 s; 28–35 cycles, PCR product size 65 bp.

All PCR reactions were initially denatured for 5 min at 95°C.

### *RFLP analysis of* ras *mutations*

(1) H-*ras* codon 12: 10–20 µl of the amplification product was digested overnight with 40 units of *Msp*I.

(2) K-*ras* codons 12 and 13: 10–20 µl of the amplification product was digested for 3 h with 20 units of *Bst*NI (codon 12) and another aliquot of 20 units of *Hph*I (codon 13, Gly to Asp).

(3) N-*ras* codon 61: 10–20 µl of the amplification product was digested overnight with 40 units of *Msc*I.

The incubation temperatures were 60°C for *Bst*NI and 37°C for the remaining enzymes. The digestion products were 354 bp and 385 bp for H-*ras* codon 12 and 113 bp and 142 bp for K-*ras* codon 12, normal and mutant alleles respectively. The normal codon 61 N-*ras* allele produced a 44 bp band, while the mutant allele remained 65 bp in size and the mutant (Gly to Asp) codon 13 K-*ras* allele produced a 115 bp band compared with a normal PCR product of 157 bp. Restriction enzymes were supplied by New England Biolabs. The digestion products were analysed on 2% agarose gels (H-*ras* codon 12, K-*ras* codon 12 and 13) or on 10% polyacrylamide gels (N-*ras* codon 61) and visualised under UV illumination after staining with ethidium bromide.

### *Competitive reverse transcription PCR (RT–PCR) and RNA quantitation*

This procedure has been described in detail elsewhere (Kotsinas *et al*., 1994). Briefly, the competitor sequence (cloned within a plasmid) has been derived by an internal deletion of the PCR product of the same set of primers, applied to genomic DNA. This results in a fragment which is smaller than the genomic DNA product, but larger than the cDNA product; thus, it shares the same primer sites and serves in the PCR reaction as a competitor. A 200 ng aliquot of total RNA was reverse transcribed in a 50 µl reaction (10 mM Tris–HCl pH 8.3, 50 mM potassium chloride, 1 mM manganese chloride, 200 mM dNTP, 200 ng of antisense primer and 2.5 units *Tth* polymerase) for 15 min. PCR amplification of cDNA was then performed by adding 50 µl of 75 mM Tris–HCl pH 9.0, 20 mM ammonium sulphate, 1.5 mM magnesium chloride, 0.01% Tween 20 (w/v), 0.75 mM EGTA and 200 ng of sense primer and using the PCR programmes previously described for 28 cycles. The number of cycles was decided after preliminary experiments showed that the PCR reaction remained in the exponential phase at this time. A standard curve was derived from serial dilutions of total RNA (0.1 µg, 0.2 µg, 0.5 µg, 1.0 µg and 2.0 µg) mixed with 50 pg of competitor plasmid (Figure 1). The PCR products were analysed in a 6% polyacrylamide gel stained with either silver or ethidium bromide. The quantity and quality of target RNA was determined by amplification of β-actin

**Figure 1** Standard curve of H-*ras* mRNA by the RT–PCR assay. Fifty picograms of competitor DNA was co-amplified with 2.0 µg (lane 1), 1.0 µg (lane 2), 0.5 µg (lane 3), 0.2 µg (lane 4) and 0.1 µg (lane 5) of total RNA. M, marker = pBR322/*Hae*III.

**Table I** Primers used for *ras* analysis

| | |
|---|---|
| H-*ras* codon 12 | 5′-GACGGAATATAAGCTGGTGG-3′<br>3′-TAACTACCCCTCTGCACGGA-5′ |
| K-*ras* codons 12 and 13 | 5′-ACTGAATATAAACTTGTGGTAGTTGGACCT-3′<br>5′-TCAAAGAATGGTCCTGGACC-3′ |
| N-*ras* codon 61 | 5′-GACATACTGGATACAGCTGGC-3′<br>5′-CCTGTCCTCATGTATTGGTC-3′ |
| VTR region | 5′-GAGCTAGCAGGGCATGCCGC-3′<br>5′-AGCACGGTGTGGAAGGAGCC-3′ |
| β-actin mRNA | 5′-GTGGGGCGCCCCAGGCACCA-3′<br>5′-CTCCTTAATGTCACGCACGATTTC-3′ |

mRNA (548 bp) using the reaction mixture described above and the following amplification conditions: 1 min at 95°C, 1 min at 58°C and 1 min at 72°C for 28 cycles. The quantity of RNA used in all experimental amplifications of H-*ras* mRNA was thus previously normalised by the prior amplification of β-actin mRNA from each sample. The interpretation of the expression levels of H-*ras* using competitive RT–PCR (using the aforementioned competitor plasmid) is calculated using the following ratio: (target *vs* competitor in the tumour tissue) to (target *vs* competitor in the normal tissue). Amplification and quantitation of H-*ras* mRNA was performed at least twice for each sample and produced similar results each time.

### Results

Among the 120 samples analysed, only two were found to contain a *ras* mutation, both of which were in codon 12 of K-*ras* (Figure 2). No mutations were found in codon 13 of K-*ras*, codon 12 of H-*ras*, or codon 61 of N-*ras*. These results are in agreement with previous studies, which show less than 5% *ras* mutations in SCCHN in the Western world (Tables II and III). As there were only two positive samples with mutations out of 120 SCCHN tested, no analysis with the clinicopathological parameters was undertaken.

Twenty-six SCCHN samples (five oropharynx, 11 hypopharynx, five oral, five larynx) were analysed for aberrant expression of H-*ras* mRNA. Twenty of the 26 SCCHN samples exhibited elevated expression of H-*ras* in the tumour tissue, ranging from 1.1-to 8.1-fold expression (Table IV, Figures 3 and 4). Since tumour cells are characterised by high rates of proliferation, small increases in the level of expression of the H-*ras* gene would be expected as being representative of a cell kinetic system in which the *ras* signal transduction pathway is activated. However, four samples (184, 360, 225 and 1092) showed slightly lower levels of the H-*ras* mRNA in their tumour tissue. Thus, we arbitrarily divided

**Figure 2** PCR–RFLP assay for the detection of K-*ras* codon 12 mutations in SCCHN. Lane 1, marker pUC18/*Hae*III; lane 2, undigested PCR product; lane 3, control DNA sample from cell line SW 480 which harbours a homozygous mutation at codon 12 of K-*ras*; lanes 4, 7 and 8, normal samples; lanes 5 and 6, mutant samples.

**Table III** Cumulative results of *ras* mutations in SCCHN in the Western world

| | *Gene and codon* | | | | | | | | |
|---|---|---|---|---|---|---|---|---|---|
| | *H*-ras | | | *K*-ras | | | *N*-ras | | |
| | *12,* | *13,* | *61* | *12,* | *13,* | *61* | *12,* | *13,* | *61* |
| *Number of samples tested* | 348 | 137 | 191 | 269 | 190 | 70 | 107 | 42 | 190 |
| *Number of mutations* | 6 | 0 | 0 | 2 | 0 | 2 | 0 | 0 | 0 |
| *Per cent mutations* | 1.7 | 0 | 0 | 0.7 | 0 | 2.8 | 0 | 0 | 0 |

**Table II** Cumulative results on *ras* mutations in SCCHN

| Ras *gene and codon* | *Number of specimens* | *Mutations (codon)* | *Reference* |
|---|---|---|---|
| *India* | | | |
| H-*ras*-12,13,61 | 57 | 8(12), 1(13), 13(61) | Saranath *et al.* (1991) |
| K-*ras*-12,13,61 | 57 | 0 | |
| N-*ras*-12,13,61 | 57 | 0 | |
| *Taiwan* | | | |
| K-*ras*-12 | 33 | 6(12) | Kuo *et al.* (1994) |
| *Western world* | | | |
| H-*ras*-12,61 | 54 | 2(12) | Sheng *et al.* (1990) |
| K-*ras*-12,13,61 | 28 | 0 | |
| N-*ras*-12,61 | 28 | 0 | |
| H-*ras*-12 | 37 | 2 | Rumsby *et al.* (1990) |
| K-*ras*-12 | 37 | 0 | |
| N-*ras*-12 | 37 | 0 | |
| K-*ras*-12 | 42 | 0 | Hirano *et al.* (1991) |
| H-*ras*-12,13,61 | 30 | 0 | Chang *et al.* (1991) |
| K-*ras*-12,13,61 | 30 | 2(61) | |
| N-*ras*-12,13,61 | 30 | 0 | |
| H-*ras*-12,13,61 | 28 | 1(12) | Warnakulasuriya *et al.* (1992) |
| H-*ras*-12,13,61 | 67 | 0 | Clark *et al.* (1993) |
| H-*ras*-12,13,61 | 12 | 1(12) | Yeudall *et al.* (1993) |
| K-*ras*-12,13,61 | 12 | 0 | |
| N-*ras*-12,13,61 | 12 | 0 | |
| H-*ras*-12 | 120 | 0 | Present study |
| K-*ras*-12,13 | 120 | 2(12) | |
| N-*ras*-61 | 120 | 0 | |

**Table IV** H-*ras* expression in SCCHN

| *Sample* | *H*-ras *mRNA levels*[a] | *VTR instability* | *Site*[b] | *Histology*[c] | *TNM stage*[d] | *Nodes at pathology* |
|---|---|---|---|---|---|---|
| 350 | 8.1 | | HP | MD | III | + |
| 338 | 7.5 | + | HP | MD | I | – |
| 355 | 6.5 | | O | WD | III | + |
| 228 | 5.1 | | OP | PD | IV | ND |
| 358 | 3.3 | – | O | MD | IV | + |
| 302 | 3.3 | + | HP | MD | III | – |
| 366 | 3.1 | | HP | WD | I | ND |
| 353 | 2.4 | | O | MD | IV | – |
| 365 | 2.2 | | OP | MD | IV | + |
| 339 | 2.2 | – | L | MD | I | – |
| 343 | 2.1 | – | O | MD | IV | + |
| 359 | 1.9 | – | L | MD | I | – |
| 351 | 1.7 | – | L | MD | IV | – |
| 218 | 1.5 | – | HP | PD | IV | + |
| 337 | 1.4 | | HP | WD | III | + |
| 336 | 1.4 | + | HP | WD | III | – |
| 192 | 1.4 | | HP | PD | IV | + |
| 1161 | 1.3 | – | O | MD | IV | ND |
| 305 | 1.2 | – | HP | MD | IV | + |
| 361 | 1.1 | | HP | MD | II | + |
| 348 | 1.1 | | OP | PD | III | + |
| 370 | 1.0 | | OP | PD | III | ND |
| 1092 | 0.9 | | OP | WD | III | – |
| 360 | 0.9 | + | L | MD | IV | + |
| 225 | 0.8 | – | L | MD | III | – |
| 184 | 0.8 | + | HP | PD | III | + |

[a]Levels of H-*ras* mRNA are expressed as the ratio of the levels in the tumour *vs* the normal tissue. [b]OP, oropharynx; HP, hypopharynx; O, oral; L, larynx. [c]WD, MD and PD, well, moderately and poorly differentiated SCC respectively. [d]TNM staging (UICC, 1978). ND, no data.

**Figure 3** Representative examples of H-*ras* overexpression in specimen numbers 228 and 353, interpreted by the RT–PCR assay. Number 370 represents a specimen with no H-*ras* overexpression, showing equal levels of expression in the normal and tumour samples. The PCR product was silver stained and scanned using the UVP image analysis system. The upper arrow indicates the competitor H-*ras* and the lower arrow the target H-*ras*.

**Figure 4** H-*ras* overexpression in tumour specimens 365, 366, 358 and 355. The PCR product was quantified using the UVP image analysis system after staining with ethidium bromide. (**a**) Competitor H-*ras*; (**b**) target H-*ras*.

the tumour samples by their H-*ras* mRNA levels of expression into two groups; patients in the first and second groups exhibited <1.5- and ≥1.5-fold expression of H-*ras*, respectively, compared with normal tissue. Thus 14 of the 26 (54%) SCCHN specimens tested showed overexpression of H-*ras* mRNA, but no association was found with site, histology, TNM staging or positive nodes at pathology (Table IV). An association was found, however, between the levels of expression of H-*ras* and the clinical outcome; 9 of the 12 patients with no evidence of H-*ras* overexpression have died, whereas 3 of the 14 patients with elevated expression of the H-*ras* proto-oncogene are dead (log-rank analysis, $\chi^2 = 4.27$, $P<0.05$). This result should be treated with caution as a number of these patients have been followed up for under 1 year (median 13 months, range 1–87 months).

PCR amplification of the 3′-VTR region was only possible in 14 of the 26 patient samples owing to technical difficulties with some of the specimens. In 5 of 14 (36%) SCCHN paired samples, a different pattern was observed in the tumour specimens compared with their normal tissue counterparts, indicating genetic instability of the VTR region. (Some of these results have been previously reported in Kiaris *et al*., 1994.) No association with clinicopathological parameters was demonstrated. An interesting observation was that, among the five SCCHN tumour specimens with VTR genetic instability, four had altered levels of H-*ras* mRNA compared with their normal tissue; two had lower levels (0.8 and 0.9) and two had higher levels (7.5 and 3.3). This may indicate that, at least in a certain number of patients, deregulation in the expression of H-*ras* is due to destabilisation of the VTR region of the gene.

### Discussion

Head and neck cancers are the sixth commonest cancers in the world but have a wide geographical variation which is most likely due to specific environmental risk factors (Vokes *et al*., 1993). This is reflected in the different incidence of *ras* mutations in the Western world compared with SE Asia and India. In the Western world, *ras* mutations in SCCHN are very rare (<5%), whereas in India 35% of SCCHN patients harbour a mutation in H-*ras*, and this has been associated with tobacco chewing (Saranath *et al*., 1991). In Taiwan, 18% of oral cancer patients investigated were found to have a K-*ras* mutation, and these patients chew betel quid but do not use tobacco (Kuo *et al*., 1994). In this study, 120 samples were analysed for mutations in codon 12 of H-*ras*, codons 12 and 13 of K-*ras* and codon 61 of N-*ras*, but only two of these samples (1.8%) contained *ras* mutations, both of which were found in codon 12 of K-*ras*. This is the largest analysis to date of *ras* mutations in SCCHN and the results confirm that *ras* mutations in SCCHN are rare in the Western world (Table III).

The expression of H-*ras* mRNA in SCCHN was investigated by competitive RT–PCR, which is a fast and sensitive technique for the interpretation of the levels of specific transcripts in cells or tissues. This is the first report to our knowledge that employs competitive RT–PCR for the investigation of H-*ras* mRNA expression in tumour tissues. In the past, Northern blot and dot blot hybridisation techniques have been used, but they have the disadvantages of being time-consuming and requiring the use of radioactivity. The primers used for H-*ras* cDNA amplification lie in adjacent exons of the H-*ras* gene, and thus it is possible to discriminate against contamination of the target RNA with genomic DNA. This particular feature of the amplification reaction and the co-amplification of the internal control (pGEM220H-ras) produces increased specificity and accuracy of results (Kotsinas *et al.*, 1994).

Significantly elevated levels of H-*ras* mRNA were found in 14 of the 26 (54%) SCCHN specimens tested, and in four (15%) patients the levels of H-*ras* mRNA were lower in the tumour than in the normal tissue. No association was found between the levels of expression of H-*ras* and the site of the tumour, TNM staging, histology or the nodal status of the patients, suggesting that, although overexpression of the H-*ras* proto-oncogene is associated with the development of SCCHN, alterations in other oncogenes or tumour-suppressor genes (TSGs) may be more important for the progression of this disease. The four TNM stage I tumours investigated in this present study all exhibited elevated levels of H-*ras* mRNA (>1.5-fold), which may indicate that the overexpression of this proto-oncogene is important in the early stages of the disease. *In vitro* studies have suggested that H-*ras* overexpression is associated with morphological transformation, but tumorigenicity is also greatly increased when it is accompanied by the overexpression of K-*ras* and N-*ras* (Coleman *et al.*, 1994). The association of H-*ras* overexpression in SCCHN with a favourable prognosis is in agreement with an earlier immunohistochemical analysis of *ras* p21 in 69 SCCHN samples (Field *et al.*, 1992).

We have previously reported LOH at the H-*ras* locus (Kiaris *et al.*, 1994). Three of the specimens that exhibited LOH were used in the present study, but no association between LOH at H-*ras* and overexpression of the gene was found. This result is in agreement with Sheng *et al.* (1990), who also demonstrated no association between overexpression of mRNA and LOH at the H-*ras* locus in 11 SCCHN specimens. However, the transcriptional activation of H-*ras* still remains a puzzle. The lack of a TATA box and the presence of a CAAT box in the GC-rich promoter at the 5′ end of the gene reveals similarities with other housekeeping genes (Breathnach and Chambon, 1981; Honkawa *et al.*, 1987). If overexpression of H-*ras* was due solely to the high proliferative status of cancer cells, then normalisation of the quantity of H-*ras* mRNA with actin mRNA (which is also a housekeeping gene) would equalise any differences between normal and tumour tissue from the same patient. However, the relative levels of expression ranged from 0.8 to 8.1, and this probably indicates that specific genetic alterations in particular tumour samples activate or inactivate H-*ras* proto-oncogene expression.

The VTR region in the 3′ end of H-*ras* possesses differential enhancer activity (Spandidos and Holmes, 1987) and may divide the SCCHN population into certain subgroups which have an increased risk of developing cancer (Krontiris *et al.*, 1993). Since we have already reported that instability occurs in a repetitive element (HRMS) located in intron 1 of H-*ras* (Kiaris *et al.*, 1994), we considered that genetic instability in the 3′-VTR region of H-*ras* may also be a possibility, thus resulting in the generation of new VTR alleles within the tumours of patients. This may explain, at least in a certain number of samples, the deregulation of H-*ras* gene. Indeed, we did find that 5/14 (36%) patients exhibited 3′-VTR instability, confirming the suggestion that the H-*ras* VTR is a potential target for instability. However, the levels of H-*ras* mRNA were not associated with instability of the 3′-VTR region; two tumours exhibited overexpression (7.5 and 3.3 times) and two had relatively lower levels of H-*ras* mRNA (0.8 and 0.9 times). This may indicate that instability of the 3′-VTR region could influence the regulation of the H-*ras* gene *in vivo*, as previously proposed after *in vitro* experiments (Green and Krontiris, 1993). However, a combined analysis of H-*ras* expression and instability of the VTR region in a larger number of patients is required to extend this observation.

Our study confirms the proposal that *ras* gene mutations are rare in SCCHN in the Western world but that overexpression of H-*ras* is associated with the development of SCCHN, however, the interpretation of this finding remains unclear. Further investigations should aim to clarify the significance of elevated transcription of the H-*ras* proto-oncogene and also to distinguish the mechanism(s) that results in the aberrant expression of H-*ras* in SCCHN. Finally, the present study illustrates the power of the competitive RT–PCR as a fast and sensitive technique for screening a relatively large number of samples for specific mRNA expression.

### References

AZUMA M, FURUMOTO N, KAWAMATA H, YOSHIDA H, YANAGAWA T, YURA Y, HAYASHI Y, TAKEGAWA Y AND SATO M. (1987). The relation of ras oncogene product p21 expression to clinicopathological status criteria and clinical outcome in squamous cell head and neck cancer. *Cancer J.*, **1**, 375–380.

BARBACID M. (1987). Ras genes. *Annu. Rev. Biochem.*, **56**, 779–827.

BOGUSKI MS AND McCORMICK F. (1993). Proteins regulating Ras and its relatives. *Nature*, **366**, 643–654.

BOS JL. (1989). Ras oncogenes in human cancer: a review. *Cancer Res.*, **49**, 4682–4689.

BREATHNACH R AND CHAMBON P. (1981). Organization and expression of eukaryotic split genes coding for proteins. *Annu. Rev. Biochem.*, **50**, 349–383.

CHANG SE, BHATIA P, JOHNSON PR, McCORMICK F, YOUNG B AND HIORNS L. (1991). Ras mutations in United Kingdom examples of oral malignancies are infrequent. *Int. J. Cancer*, **48**, 409–412.

CLARK LJ, EDINGTON K, SWAN IR, McLAY KA, NEWLANDS WJ, WILLS LC, YOUNG HA, JOHNSTON PW, MITCHELL R, ROBERTSON G AND OTHERS. (1993). The absence of Harvey ras mutations during development and progression of squamous cell carcinomas of the head and neck. *Br. J. Cancer*, **68**, 617–620.

COLEMAN WB, THRONEBURG DB, GRISHAM JW AND SMITH GJ. (1994). Overexpression of c-K-ras, c-N-ras and transforming growth factor β co-segregate with tumorigenicity in morphologically transformed C3H 10T1/2 cell lines. *Carcinogenesis*, **15**, 1005–1012.

FIELD JK. (1992). Oncogenes and tumour-suppressor genes in squamous cell carcinoma of the head and neck. *Eur. J. Cancer Oral Oncol.*, **28B**, 67–76.

FIELD JK AND SPANDIDOS DA. (1990). The role of ras and myc oncogenes in human solid tumours and their relevance in diagnosis and prognosis. A revew. *Anticancer Res.*, **10**, 1–22.

FIELD JK, LAMOTHE A AND SPANDIDOS DA. (1986). Clinical relevance of oncogene expression in head and neck tumours. *Anticancer Res.*, **6**, 595–600.

FIELD JK, YIAGNISIS M, SPANDIDOS DA, GOSNEY JR, PAPADIMITRIOU K, VAUGHAN ED AND STELL PM. (1992). Low leves of ras p21 oncogene expression correlates with clinical outcome in head and neck squamous cell carcinoma. *Eur. J. Surg. Oncol.*, **18**, 168–176.

FOLEY KP, LEONARD MW AND ENGEL JD. (1993). Quantitation of RNA using the polymerase chain reaction. *Trends Genet.*, **9**, 380–385.

GARCIA I, DIETRICH P-Y, AAPRO M, VAUTHIER G, VADAS L AND ENGEL E. (1989). Genetic alterations of c-myc, c-erbB-2, and c-Ha-ras-1 protooncogenes and clinical associations in human breast carcinomas. *Cancer Res.*, **49**, 6675–6679.

GREEN M AND KRONTIRIS TG. (1993). Allelic variation of reporter gene activation by the HRAS1 minisatellite. *Genomics*, **17**, 429–434.

HIRANO T, STEELE PE AND GULCKMAN JL. (1991). Low incidence of point mutation at codon 12 of K-ras proto-oncogene in squamous cell carcinoma of the upper aerodigestive tract. *Ann. Otol. Rhinol. Laryngol.*, **100**, 597–599.

HONKAWA H, MASAHASHI W, HASHIMOTO S AND HASHIMOTO-GOTOH T. (1987). Identification of the principal promoter sequence of the c-H-ras transforming oncogene: deletion analysis of the 5′-flanking region by focus formation assay. *Mol. Cell Biol.*, **7**, 2933–2940.

HONKAWA H, MASAHASHI W, HASHIMOTO S AND HASHIMOTO-GOTOH T. (1987). Identification of the principal promoter sequence of the c-H-ras transforming oncogene: deletion analysis of the 5'-flanking region by focus formation assay. *Mol. Cell Biol.*, **7,** 2933-2940.

HOWELL RE, WONG FS AND FENWICK RG. (1989). Loss of Harvey ras heterozygosity in oral squamous carcinoma. *J. Oral Pathol.*, **18,** 79-83.

KIARIS H, SPANDIDOS DA, JONES AS AND FIELD JK. (1994). Loss of heterozygosity and microsatellite instability of the H-ras gene in cancer of the head and neck. *Int. J. Oncol.*, **5,** 579-582.

KOTSINAS A, KIARIS H AND SPANDIDOS DA. (1994). A method to detect and quantitate the expression of normal versus mutant H-ras transcripts at codon 12. *Int. J. Oncol.*, **5,** 479-483.

KRONTIRIS TG, DEVLIN B, KARP DD, ROBERT NJ AND RISCH N. (1993). An association between the risk of cancer and mutations in the HRAS1 minisatellite locus. *N. Engl. J. Med.*, **329,** 517-523.

KUO MYP, JENG JH, CHIANG CP AND HAHN LJ. (1994). Mutations of Ki-ras oncogene codon 12 in betel quid chewing-related human oral squamous cell carcinoma in Taiwan. *J. Oral. Pathol. Med.*, **23,** 70-74.

RUMSBY G, CARTER RL AND GUSTERSON BA. (1990). Low incidence of ras-oncogene activation in human squamous cell carcinomas. *Br. J. Cancer*, **61,** 365-368.

SARANATH D, CHANG SE, BHOITE IT, PANCHAL RG, KERR IB, MEHTA AR, JOHNSON NW AND DEO MG. (1991). High frequency mutation in codons 12 and 61 of H-ras oncogene in chewing tobacco-related human oral carcinoma in India. *Br. J. Cancer*, **63,** 573-578.

SCAMBIA G, CATOZZI L, BENEDETTI PANICI P, FERRANDINA G, ALMADORI G, PALUDETTI G, CADONI G, DISTEFANO M, PIFFANELLI A, MANCUSO S AND MAURIZI M. (1994). Expression of ras oncogene p21 protein in normal and neoplastic laryngeal tissues: correlation with histopathological features and epidermal growth factor receptors. *Br. J. Cancer*, **69,** 959-999.

SHENG ZM, BARROIS M, KLIJANIENKO J, MICHEAU C, RICHARD JM AND RIOU G. (1990). Analysis of the c-Ha-ras-1 gene for deletion, mutation, amplification and expression in lymph node metastases of human head and neck carcinomas. *Br. J. Cancer*, **62,** 398-404.

SHIRAISHI M, MORINAGA S, NOGUCHI M, SHIMOSATO Y AND SEKIYA T. (1987). Loss of genes on the short arm of chromosome 11 in human lung carcinomas. *Jpn J. Cancer Res.*, **78,** 1302-1308.

SPANDIDOS DA, FRAME M AND WILKIE NM. (1990). Expression of the normal H-ras1 gene can suppress the transformed and tumorigenic phenotypes induced by mutant ras genes. *Anticancer Res.*, **10,** 1543-1554.

SPANDIDOS DA AND HOLMES L. (1987). Transcriptional enhancer activity in the variable tandem repeat DNA sequence downstream of the human Ha-ras-1 gene. *FEBS Lett.*, **218,** 41-46.

SPANDIDOS DA AND WILKIE NM. (1984). Malignant transformation of early passage rodent cells by a single mutated human oncogene. *Nature*, **310,** 469-475.

SPANDIDOS DA, LAMOTHE A AND FIELD JK. (1985). Multiple transcriptional activation of cellular oncogenes in human head and neck solid tumours. *Anticancer Res.*, **5,** 221-224.

TSUJI T, SASAKI K, HIRAOKA F AND SHINOZAKI F. (1989). The immunohistochemical detection of ras p21 and its correlation with differentiation in oral cancers. *J. Tumour Marker Oncol.*, **4,** 415-419.

UICC. (1978). TNM Classification of Malignant Tumours, Harmer MN. (ed.) UICC: Geneva.

VACHTENHEIM J, HORAKOVA I AND NOVOTNA H. (1994). Hypomethylation of CCGG sites in the 3'-region of H-ras protooncogene is frequent and is associated with H-ras allele loss in non-small cell lung cancer. *Cancer Res.*, **54,** 1145-1148.

VOKES EE, WEICHSELBAUM RR, LIPPMAN SM AND HONG WK. (1993). Head and neck cancer. *N. Engl. J. Med.*, **328,** 184-194.

WARNAKULSURIYA KAAS, CHANG SE AND JOHNSON NW. (1992). Point mutations in the Ha-ras oncogene are detectable in formalin-fixed tissues of oral squamous cell carcinomas, but are infrequent in British cases. *J. Oral. Pathol. Med.*, **21,** 225-229.

YEUDALL WA, TORRANCE LK, ELSEGOOD KA, SPEIGHT P, SCULLY C AND PRIME SS. (1993). ras Gene point mutation is a rare event in premalignant tissues and malignant cells and tissues from oral mucosal lesions. *Eur. J. Cancer Oral Oncol.*, **29B,** 63-67.